# Effect of prescribing metformin according to eGFR instead of serum creatinine level: A study based on Korean National Health and Nutrition Examination Survey (KNHANES) 2009-2014

**DOI:** 10.1371/journal.pone.0175334

**Published:** 2017-04-11

**Authors:** Sun Joon Moon, Chang Ho Ahn, Young Min Cho

**Affiliations:** Department of Internal Medicine, Seoul National University College of Medicine, Seoul, Republic of Korea; The University of Tokyo, JAPAN

## Abstract

**Background:**

The metformin label has recently been changed from serum creatinine (sCr)-based to estimated glomerular filtration rate (eGFR)-based indication, which is expected to expand its use for patients with mild renal insufficiency. However, because the sCr level is lower in Asians than in Caucasians at the same level of renal function, this change might not expand metformin use in the Asian population. We investigated the effect of this change among Korean patients with diabetes.

**Methods:**

Data from the Korean National Health and Nutrition Examination Survey 2009 to 2014 were used and included 4,127 adult patients with diabetes. The metformin eligibility was assessed by the sCr level (1.4 mg/dL for women and 1.5 mg/dL for men) or by eGFR categories (contraindicated, <30; indeterminate, ≥30, <45; likely safe, ≥45 mL/min/1.73 m^2^) calculated by various eGFR equations including MDRD equation. We designated the ‘expanding’ and ‘contracting’ population as those who are likely safe according to eGFR among sCr-ineligible patients and those contraindicated according to eGFR among sCr-eligible patients, respectively. Results were weighted to the whole Korean adult population.

**Results:**

All eGFR equations showed expansion in the population for whom metformin is likely safe, ranging from 14.3% to 19.9% of the sCr-ineligible population. With the MDRD equation, the expanding population was 15,264 (15.8%) and the contracting population was 0 (0.0%). Male sex and younger age were significantly associated with the expanding population.

**Conclusions:**

Contrary to our concern, prescribing metformin according to eGFR substantially expanded the indication of its use among the Korean diabetic patients.

## Introduction

Metformin, a biguanide, was first introduced to clinical practice in the 1950s and the drug’s benefits are supported by numerous clinical studies. Therefore, metformin has been used to treat type 2 diabetes as the first-line oral anti-diabetes drug (OAD) [1]. However, Phenformin, an earlier biguanide, was banned from the market in 1977, because of its devastating side effect namely lactic acidosis, which was associated with more than 50% mortality [2, 3]. Furthermore, there has been a concern that, as a biguanide, metformin would also be associated with this fatal complication. Because metformin is mainly eliminated through the kidney [4], it was believed that decreased renal function followed by accumulation of the drug might result in lactic acidosis. Subsequently, metformin has been contraindicated among patients with renal insufficiency as indicated by serum creatinine (sCr) ≥ 1.4 mg/dL in women and ≥ 1.5 mg/dL in men. These cutoff thresholds were determined by the renal function with which 3 g of metformin could be removed over 24 to 48 h [5], and the United States Food and Drug Administration (FDA) approved the use of metformin with the above contraindication in 1994 [6]. However, the incidence of lactic acidosis among metformin users was very low, with figures as low as 2.4 to 9.0 per 100,000 patient-years [7], whereas that of phenformin was 40 to 64 cases per 100,000 patient-years [8]. Moreover, some studies revealed that the incidences of the complication were not different among users of metformin and other OADs [9]. On the basis of these findings, it was suggested that lactic acidosis was not significantly associated with metformin [10], and even if the complication occurred, fatality was relatively less than with other drugs [11].

Considering metformin’s remarkable beneficial effects and rare side effects, it has been argued that its restriction regarding renal insufficiency should be relieved. Many associations tried to solve this problem by changing the prescription guideline from an sCr-based model to an estimated glomerular filtration rate (eGFR)-based model [12–15]. According to a US study that used data from the National Health and Nutrition Examination Surveys (NHANES) [16], it has been estimated that by applying this change, 14.6% more patients with diabetes might be able to use metformin, who previously were contraindicated by the sCr level. Recently, the FDA has changed the label of metformin as follows: “metformin is contraindicated in patients with an eGFR below 30 mL/min/1.73 m^2^ and starting metformin in patients with an eGFR between 30–45 mL/min/1.73 m^2^ is not recommended” [17].

Intriguingly, previous studies have revealed that Asians have relatively lower sCr levels than Caucasians with the same degree of renal function. Serum creatinine concentrations corresponding to measured GFR (inulin clearance) of 60 ml/min/1.73 m^2^ were 1.55 mg/dL sCr in men and 1.18 mg/dL in women for Caucasians [18], whereas in Japanese subjects, it corresponded to 1.04 mg/dL sCr in men [19]. With other methods, the measured GFR (creatinine clearance) of 60 ml/min/1.73 m^2^ corresponded to the sCr level of 1.15 mg/dL in men and 0.95 mg/dL in women among Korean patients with type 2 diabetes [20] and 0.91 mg/dL in men and 0.73 mg/dL in women in Pakistani subjects [21]. These findings all agree on the point that Asians have lower GFR levels at the same values of sCr compared to Caucasians.

Consequently, we were concerned that when applying the eGFR method instead of sCr, the expansion of metformin indication seen in the U.S. population would decline, or the indication would even contract in the Asian population. In addition, as mentioned above, the study using NHANES data was composed mainly of White, Non-Hispanic Black, and Hispanic subjects, and only a few Asians were included in the study [16]. This leaves room for uncertainty on whether using this method for Asian patients would show similar results. Subsequently, the aim of the present study was to examine the effect on metformin treatment indication among the Korean diabetic population, by applying eGFR-based prescription instead of sCr-based.

## Materials and methods

### Study design

The present study was performed using the data of the Korea National Health and Nutrition Examination Survey (KNHANES), which was conducted by Korea Centers for Disease Control and Prevention (KCDC) to evaluate the health and nutritional status of the non-institutionalized civilian population in Korea since 1998 [22, 23]. This survey is a nationwide cross-sectional study involving a sampling plan that follows a multistage clustered probability design to enable analysis of the data from the total national population [23, 24]. Trained medical staff and interviewers carried out the health interview, health examination, and nutrition survey to obtain KNHANES information. The study protocol was approved by KCDC institutional review board (2009-01CON-03-2C, 2010-02CON-21-C, 2011-02CON-06-C, 2012-01EXP-01-2C, 2013-07CON-03-4C, 2013-12EXP-03-5C). All participants in the survey signed informed consents.

### Study population

**Fig 1:**
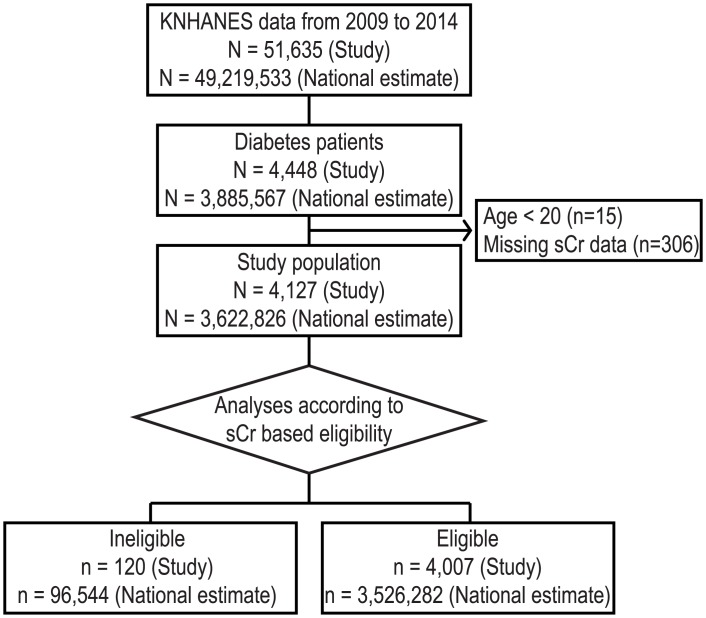
Flow diagram of study selection. KNHANES, Korea National Health and Nutrition Examination Survey; sCr, serum creatinine. “Study” refers to actual study participants’ data. “National estimate” refers to representative population estimates of the total Korean population.

Participants of KNHANES from 2009 to 2014 were included in this study, which was the most recent data available. During this period, studies had been conducted using the same method to measure sCr. We included only patients with diabetes, defined as individuals 1) who were previously diagnosed with diabetes by answering interview questions, 2) whose fasting plasma glucose was 126 mg/dL or more, 3) whose HbA1c level was 6.5% (48 mmol/mol) or more, or 4) who were on insulin or OADs. We excluded participants who were under 20 years old, who were pregnant, or whose sCr data were unavailable (Fig 1).

### Definitions

Definitions used in the present study are analogous to a previously mentioned study regarding NHANES data [16]. According to past FDA labeling, “ineligible by sCr” was defined as sCr ≥1.4 mg/dL for women and ≥1.5 mg/dL for men and “eligible by sCr”, <1.4 mg/dL for women and <1.5 mg/dL for men, respectively. Following the recommendations by many associations [12, 13, 15, 16], we used definitions of eGFR categories as follows: contraindicated, <30 mL/min/1.73 m^2^; indeterminate, ≥30, <45 mL/min/1.73 m^2^; likely safe, ≥45 mL/min/1.73 m^2^.

Equations used for eGFR or creatinine clearance are as follows: 1) 4-variable Modification of Diet in Renal Disease (MDRD) equation for calibrated sCr; 175 x (S_cr_)^-1.154^ x (Age)^-0.203^ x (0.742 if female) x (1.212 if African American) [25], 2) Chronic Kidney Disease Epidemiology Collaboration (CKD-EPI) equation; 141 x min (S_cr_ /κ,1)^α^ x max (S_cr_ /κ, 1)^-1.209^ x 0.993^Age^ x 1.018 (if female) x 1.159 (if black) [26], 3) Cockcroft-Gault (CG) equation; (140—age) x lean body weight / (S_cr_ x 72) x 0.85 (if female) [27], 4) CG equation corrected for body surface area (BSA); CG equation/ (1.73 x BSA). In the CKD-EPI equation, α and κ refer to coefficients and min and max to functions, and the details have been previously published [26]. We used actual body weight instead of lean body weight for CG, and BSA was calculated by the following equation: 0.007184 x weight^0.425^ x height^0.725^ [28]. The *CG equation corrected for BSA* was marked as *CG/ 1*.*73 m*^*2*^ in this study, for convenience.

In this study, we defined the expanding population as a likely safe group according to the eGFR-based method in the sCr-ineligible group. However, according to recent FDA labeling, newly prescribed metformin is not recommended for the eGFR-indeterminate group, leading to the decision to exclude this group from the expanding population. Also, we defined the contracting population as contraindicated individuals when the eGFR method was used in the sCr eligible group. The difference between the expanding population and the contracting population was regarded as the net expanding population.

Finally, individuals with hypertension were defined as having mean measured systolic blood pressure ≥ 140 mm Hg or diastolic blood pressure ≥ 90 mm Hg, or currently taking any anti-hypertensive medications.

### Measurements

Information on age, sex, history of diagnosis (diabetes, hypertension), and medication history were obtained by self-report questionnaires. Body weight, height, and blood pressure were measured during physical examination, and body mass index (BMI) was calculated using body weight and height data. Serum creatinine concentration was measured by the modified kinetic method of Jaffe using a Hitachi 7600 analyzer (Hitachi Co., Tokyo, Japan), and the coefficient of variation was within 5%. In addition, sCr data were calibrated to isotope dilution mass spectrometry (IDMS). Levels of HbA1c were measured using high-performance liquid chromatography.

### Statistical analysis

Categorical variables are demonstrated as number and proportion, and continuous variables are shown as mean ± standard deviation (SD) or standard error (SE). We analyzed baseline characteristics of the population according to sCr-based eligibility, using *t*-test and χ^2^ test. Equations for eGFR were applied among the sCr-based ineligible and eligible group, and corresponding populations were analyzed. To identify independent factors associated with the expanding population, we performed a multivariable logistic regression analysis including age, sex, BMI, hypertension, and HbA1c as covariates. Age, BMI, and HbA1c were considered as linear continuous variables.

Every analysis was presented with the national estimate data that represents the total national population by using clusters, strata, and weights [23]. For the results and statistical analyses, we used national estimate data unless otherwise mentioned. Statistical analyses were executed using SPSS software package, version 19.0 (IBM Corp, Armonk, NY). *P*-value of less than 0.05 was regarded as statistically significant.

## Results

### Baseline characteristics

A total of 51,635 subjects participated in the survey of the KNHANES study (national estimate N = 49,219,533), among whom 4,448 were patients with diabetes (national estimate N = 3,885,567). After implementation of the selection procedures, the study population was 4,127 (national estimate N = 3,622,826). When the study population was categorized using the sCr level, 2.7% were ineligible for metformin treatment (study n = 120, national estimate n = 96,544) and 97.3% (study n = 4,007, national estimate n = 3,526,282) were eligible (Fig 1, Table 1).

**Table 1:** Baseline characteristics of total study population according to metformin eligibility by sCr level, KNHANES 2009–2014.

N (%)	Ineligible by serum creatinine		Eligible by serum creatinine		*P*^b^ (Study)	*P*^b^ (National estimate)
	sCr ≥1.4 mg/dL for women;		sCr <1.4 mg/dL for women;			
	≥1.5 mg/dL for men		<1.5 mg/dL for men			
	Study^a^	National estimate^a^	Study^a^	National estimate^a^		
	120 (2.9)	96,544 (2.7)	4,007 (97.1)	3,526,282 (97.3)		
Male sex, n (%)	90 (75.0)	72,703 (75.3)	1,986 (49.6)	1,950,243 (55.3)	<.001	0.001
Age, years	67.2 ± 9.6	63.8 ± 1.3	61.8 ± 12.0	58.4 ± 0.3	<.001	<.001
Hypertension, n (%)	89 (74.2)	74,411 (77.1)	2,310 (57.6)	1,850,940 (52.5)	<.001	<.001
HbA1C, % (mmol/mol)	7.5 ± 1.6 (58 ± 17.5)	7.5 ± 0.2 (58 ± 2.2)	7.3 ± 1.4 (56 ± 15.3)	7.3 ± 0.0 (56 ± 0.0)	0.080	0.509
BMI, kg/m^2^	24.7 ± 3.1	24.9 ± 0.4	25.2 ± 3.6	25.3 ± 0.1	0.121	0.273
MDRD eGFR, mL/min/1.73 m^2^	34.0 ± 10.1	33.5 ± 1.3	82.7 ± 18.1	84.5 ± 0.4	<.001	<.001
Serum Creatinine, mg/dL	2.20 ± 1.78	2.43 ± 0.32	0.85 ± 0.19	0.86 ± 0.00	<.001	<.001
0-<1 mg/dL, n (%)	0 (0.0)	0 (0.0)	3,235 (80.7)	2,815,356 (79.8)	<.001	<.001
1-<2 mg/dL, n (%)	79 (65.8)	59,666 (61.8)	772 (19.3)	710,926 (20.2)	<.001	<.001
2-<3 mg/dL, n (%)	32 (26.7)	27,837 (28.8)	0 (0.0)	0 (0.0)	<.001	<.001
≥3 mg/dL, n (%)	9 (7.5)	9,041 (9.4)	0 (0.0)	0 (0.0)	<.001	<.001

Values for categorical variables are presented as n (%); for continuous variables, as mean ± standard deviation (for study data) or standard error (for national estimate data).

^a^ “Study” refers to actual study participants’ data. “National estimate” refers to representative population estimates of the total Korean population.

^b^*P*(study) and *P*(National estimate) are from the comparisons between the ineligible and eligible subjects from the actual study population and the estimated total Korean population, respectively.

The baseline characteristics were compared between the 2 groups (Table 1). The ineligible group had more male, aged and hypertensive subjects than the eligible group. The level of HbA1c and the BMI did not show a significant difference between the 2 groups. The mean MDRD eGFR and sCr level in the ineligible group were 33.5 ml/min/1.73 m^2^ and 2.43 mg/dL, respectively, whereas those of the eligible group were 84.5 mL/min/1.73 m^2^ and 0.86 mg/dL, respectively.

### Change in the eligibility when eGFR equation was used

We analyzed the change in patient eligibility when the eGFR-based data were used instead of the sCr levels, which was the aim of the current study. When the eGFR was used in the sCr-ineligible group, the likely safe (≥45 mL/min/1.73 m^2^ or mL/min) population was 15.8% (study n = 16, national estimate n = 15,264) with MDRD, 14.4% (study n = 14, national estimate n = 13,901) with CKD-EPI, 19.9% (study n = 20, national estimate n = 19,247) with CG, and 14.3% (study n = 13, national estimate n = 13,806) with CG/ 1.73 m^2^. When the eGFR was used, the expansion of the eligible population ranged from a minimum of 13,806 (14.3% of the sCr ineligible group, CG/ 1.73 m^2^) to a maximum of 19,247 (19.9%, CG).

**Fig 2:**
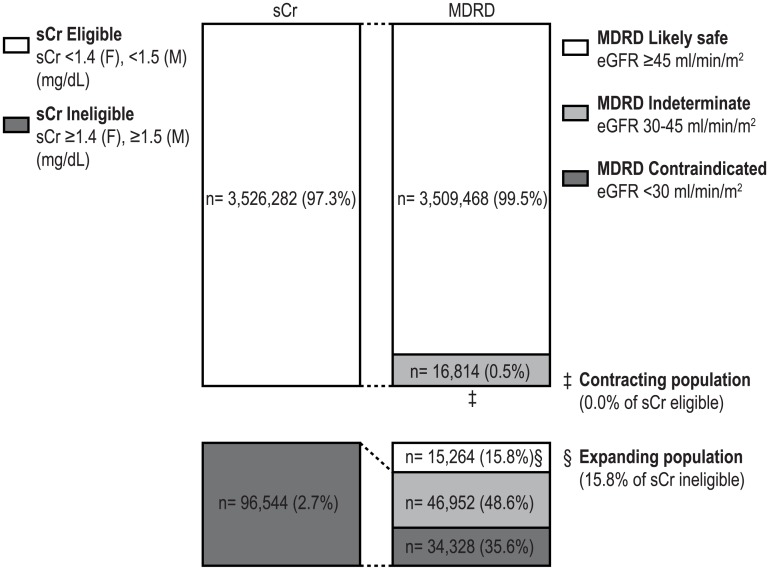
Expanding effects of changing from sCr-based to eGFR-based (MDRD) metformin prescription. Each number represents national estimates of each populations. sCr, serum creatinine; eGFR, estimated glomerular filtration rate; F, female; M, male.

When eGFR was used in the sCr-eligible group, no contraindicated population was found with MDRD and CKD-EPI, 0.26% (study n = 16, national estimate n = 9,257) with CG, and 0.02% (study n = 2, national estimate n = 827) with CG/ 1.73 m^2^. When eGFR was used, the eligible population contraction was close to 0%, although CG resulted in the highest cases of contraindication, which amounted to 9,257 (0.26% of the sCr eligible group) (Table 2, Fig 2).

**Table 2:** eGFR categories of ineligible and eligible population by the sCr level, KNHANES 2009–2014.

Ineligible by sCr (sCr ≥1.4 mg/dL for women, ≥1.5 mg/dL for men)								
	MDRD		CKD-EPI		CG^b^		CG/ 1.73 m^2^ ^c^	
	Study^a^	National estimate^a^	Study^a^	National estimate^a^	Study^a^	National estimate^a^	Study^a^	National estimate^a^
eGFR, mL/min/1.73 m^2^	34.0 ± 10.1	33.5 ± 1.3	34.3 ± 10.5	34.1 ± 1.4	33.9 ± 12.2	34.8 ± 1.6	34.3 ± 10.3	35.0 ± 1.4
eGFR <30, n (%)	36 (30.0)	34,328 (35.6)	38 (31.7)	35,251 (36.5)	50 (41.7)	37,114 (38.4)	32 (26.7)	24,015 (24.9)
eGFR ≥30, <45, n (%)	68 (56.7)	46,952 (48.6)	68 (56.7)	47,392 (49.1)	50 (41.7)	40,183 (41.6)	75 (62.5)	58,723 (60.8)
eGFR ≥45, n (%) (Expanding population)	16 (13.3)	15,264 (15.8)	14 (11.7)	13,901 (14.4)	20 (16.7)	19,247 (19.9)	13 (10.8)	13,806 (14.3)
Total ineligible population, n (%)	120 (100.0)	96,544 (100.0)	120 (100.0)	96,544 (100.0)	120 (100.0)	96,544 (100.0)	120 (100.0)	96,544 (100.0)
Eligible by sCr (sCr <1.4 mg/dL for women, <1.5 mg/dL for men)								
	MDRD		CKD-EPI		CG^b^		CG/ 1.73 m^2^ ^c^	
	Study^a^	National estimate^a^	Study^a^	National estimate^a^	Study^a^	National estimate^a^	Study^a^	National estimate^a^
eGFR, mL/min/1.73 m^2^	82.7 ± 18.1	84.5 ± 0.4	85.1 ± 16.4	87.8 ± 0.3	80.5 ± 28.0	86.2 ± 0.6	82.0 ± 23.7	86.2 ± 0.5
eGFR <30, n (%) (Contracting population)	0 (0.0)	0 (0.0)	0 (0.0)	0 (0.0)	16 (0.4)	9,257 (0.3)	2 (0.1)	827 (0.02)
eGFR ≥30, <45, n (%)	27 (0.7)	16,814 (0.5)	25 (0.6)	15,419 (0.4)	222 (5.6)	165,550 (4.7)	112 (2.8)	76,568 (2.2)
eGFR ≥45, n (%)	3,980 (99.3)	3,509,468 (99.5)	3,982 (99.4)	3,510,863 (99.6)	3,760 (94.0)	3,345,530 (95.0)	3,881 (97.1)	3,439,795 (97.8)
Total eligible population, n (%)	4,007 (100.0)	3,526,282 (100.0)	4,007 (100.0)	3,526,282 (100.0)	3,998 (100.0)	3,520,337 (100.0)	3,995 (100.0)	3,517,190 (100.0)
Net expanding population, n (%) (Expanding population -Contracting population)	16 (0.39)	15,264 (0.42)	14 (0.34)	13,901 (0.38)	4 (0.10)	9,990 (0.28)	11 (0.27)	12,979 (0.36)
Total diabetic population, n (%)	4,127 (100.0)	3,622,826 (100.0)	4,127 (100.0)	3,622,826 (100.0)	4,118 (100.0)	3,616,881 (100.0)	4,115 (100.0)	3,613,734 (100.0)

Values for categorical variables are presented as n (%); for continuous variables as mean ± standard deviation (for study data) or standard error (for national estimate data).

^a^“Study” refers to actual study participants’ data. “National estimate” refers to representative population estimates of the total Korean population.

^b^Cockroft-Gault (CG) equation uses mL/min. The total sCr-eligible population among CG equation is 3,998, because of missing body weight data.

^c^CG/1.73 m^2^ is defined as CG equation corrected for body surface area. The total sCr-eligible population among CG/1.73 m^2^ is 3,995 because of missing body weight and height data.

The net expanding population, defined as the difference between the expanding population and the contracting population in the whole study population (either eligible or ineligible for metformin use), was found to be 15,264 with MDRD (0.42% of the adult diabetic population), 13,901 with CKD-EPI (0.38%), 9,990 with CG (0.28%), and 12,979 with CG/1.73 m^2^ (0.36%). This shows that the use of metformin is indicated in significantly more patients with diabetes when eGFR is used.

### Characteristics of expanding and contracting populations

The expanding population was analyzed using MDRD and CKD-EPI, which are 2 of the most commonly used eGFR equations. Multivariable logistic regression was used and was adjusted for sex, age, hypertension, HbA1c, and BMI (Table 3). Adjusted odds ratio [aOR] was calculated to identify the odds of being in the expanded population. In both equations used, the expanding population (eGFR ≥45 mL/min/1.73 m^2^ among sCr-ineligible) had more male sex and younger age patients than the rest of the population (<45 mL/min/1.73 m^2^ among sCr-ineligible). The logistic regression analysis with sex could not be applied because the expanding population was 100% male. In regard to age, aOR was 0.92 [95% CI 0.85–0.99] for MDRD equation. Hypertension, HbA1c, and BMI did not show any significant difference in the expanding population.

**Table 3:** Characteristics according to eGFR categories of ineligible population by the sCr level, KNHANES 2009–2014.

N (%)	MDRD eGFR <45 mL/min/1.73 m^2^		MDRD eGFR ≥45 mL/min/1.73 m^2^ (Expanding population)		Adjusted OR (95% CI)^b^ (Study)	*P*^b^ (Study)	Adjusted OR (95% CI)^b^ (National estimate)	*P*^b^ (National estimate)
	Study^a^	National estimate^a^	Study^a^	National estimate^a^				
	104 (86.7)	81,280 (84.2)	16 (13.3)	15,264 (15.8)				
Male sex, n (%)	74 (71.2)	57,438 (70.7)	16 (100.0)	15,264 (100.0)	N/A^c^	N/A^c^	N/A^c^	N/A^c^
Age, years	68.2 ± 8.9	65.1 ± 1.3	60.1 ± 11.0	56.9 ± 3.0	0.91 (0.86–0.97)	0.006	0.92 (0.85–0.99)	0.031
Hypertension, n (%)	77 (74.0)	62,262 (76.6)	12 (75.0)	12,149 (79.6)	0.78 (0.19–3.23)	0.731	0.89 (0.25–3.17)	0.854
HbA1C, % (mmol/mol)	7.6 ± 1.5 (60 ± 16.4)	7.5 ± 0.2 (58 ± 2.2)	7.4 ± 1.8 (57 ± 19.7)	7.4 ± 0.5 (57 ± 5.5)	0.94 (0.64–1.37)	0.741	0.98 (0.66–1.47)	0.933
BMI, kg/m^2^	24.6 ± 3.1	24.8 ± 0.5	25.5 ± 3.2	25.2 ± 0.6	1.09 (0.90–1.33)	0.385	1.03 (0.84–1.26)	0.791
Serum Creatinine, mg/dL	2.30 ± 1.89	2.59 ± 0.38	1.54 ± 0.06	1.55 ± 0.02	-	<.001^d^	-	<.001 ^d^
N (%)	CKD-EPI eGFR <45 mL/min/1.73 m^2^		CKD-EPI eGFR≥45 mL/min/1.73 m^2^ (Expanding population)		Adjusted OR (95% CI)^b^ (Study)	*P*^b^ (Study)	Adjusted OR (95% CI)^b^ (National estimate)	*P*^b^ (National estimate)
	Study^a^	National estimate^a^	Study^a^	National estimate^a^				
	106 (88.3)	82,643 (85.6)	14 (11.7)	13,901 (14.4)				
Male sex, n (%)	76 (71.7)	58,802 (71.2)	14 (100.0)	13,901 (100.0)	N/A^c^	N/A^c^	N/A^c^	N/A^c^
Age, years	68.4 ± 8.9	65.3 ± 1.3	57.8 ± 9.5	54.9 ± 2.6	0.87 (0.80–0.95)	0.001	0.88 (0.81–0.95)	0.002
Hypertension, n (%)	79 (74.5)	63,626 (77.0)	10 (71.4)	10,785 (77.6)	0.55 (0.11–2.72)	0.462	0.67 (0.16–2.83)	0.582
HbA1C, % (mmol/mol)	7.6 ± 1.5 (60 ± 16.4)	7.5 ± 0.2 (58 ± 2.2)	7.4 ± 2.0 (57 ± 21.9)	7.4 ± 0.6 (57 ± 6.6)	0.94 (0.63–1.39)	0.743	1.00 (0.68–1.45)	0.985
BMI, kg/m^2^	24.7 ± 3.2	24.9 ± 0.5	24.8 ± 2.8	24.8 ± 0.6	0.98 (0.78–1.22)	0.840	0.96 (0.79–1.16)	0.659
Serum Creatinine, mg/dL	2.29 ± 1.88	2.58 ± 0.37	1.54 ± 0.06	1.55 ± 0.02	-	<.001 ^d^	-	<.001 ^d^

Values for categorical variables are presented as n (%); for continuous variables, as mean ± standard deviation (for study data) or standard error (for national estimate data).

^a^“Study” refers to actual study participants’ data. “National estimate” refers to representative population estimates of the total Korean population.

^b^Logistic regression analysis adjusted for sex, age, hypertension, HbA1c and BMI was performed. Age, BMI and HbA1c were modeled as linear variables.

^c^Because there is no female participant in eGFR ≥45 group among the sCr-ineligible population, logistic regression data for sex was not available.

^d^Serum creatinine data were not included in logistic regression analysis, and Kruskal-Wallis test was used to evaluate *P* value.

## Discussion

It is worthwhile to compare the expanding effect of the eGFR-based model with that of the NHANES study of the U.S. population. When MDRD eGFR was used, the expanding effect of the US population was 14.6% (for actual study data) and 11.2% (for national estimate data) of the sCr-ineligible population [16]. The present study showed similar results, with 13.3% and 15.8%, respectively (S1 Table). In addition, the contracting effect was 0% in both MDRD and CKD-EPI eGFR in both the US and the Korean population. This implies that metformin indication does not contract when eGFR is used. Moreover, it signifies that the currently used sCr-based model is indeed safe.

The present study was executed because Asians have a relatively lower sCr level for a given renal function [18–21], and therefore, we were concerned that when eGFR was used, the expansion of metformin indication previously shown in the US population would diminish, or the indication would even contract in the Asian population. However, contrary to our concern, metformin indication expanded in all the models of eGFR equation compared to the sCr-based model.

The reason why the present study did not significantly differ from the US studies when MDRD or CKD-EPI was used can be explained by inspecting the variables in each equation. The variables in MDRD or CKD-EPI include sCr level, age, sex, and ethnicity, whereas body weight is excluded from the equation. It has been proposed that Asians have lower sCr compared with Caucasians because they have a relatively low fat-free mass [20]. Since body weight is not included in the MDRD or CKD-EPI equations, the results from Asian subjects are expected not to differ significantly from the studies of US population. On the other hand, body weight is one of the variables of the CG equation, and its net expanding population was lower than that of other eGFR equations. This is compatible with the ideas previously alluded to (Table 2).

The present study is considered conservative in that only the eGFR measures of likely safe groups were included in the expanding population. If more liberal measures were used and the indeterminate group were also considered, hence allowing metformin indication in patients with eGFR ≥30 mL/min/1.73 m^2^, then the following results would ensue. The net effect would be expanded to include 62,216 subjects using MDRD (1.72% of total diabetes population), 61,293 using CKD-EPI (1.69%), 50,173 using CG (1.39%), and 71,702 using CG/1.73 m^2^ (1.98%). This shows that the indication would be widely expanded in general.

Thorough analysis of the characteristics of the expanding population revealed that when the eGFR-based model was used, metformin indication was expanded largely in male sex and younger age patients. In this regard, young-onset type 2 diabetes (YOD) refers to type 2 diabetes diagnosed before age 40 years, which is characterized by poor glycemic control and a higher rate of complications including cardiovascular disease [29, 30]. In addition, studies showed that the YOD group was associated with male sex, obesity, family history of diabetes, alcohol use, and smoking [31]. Data obtained from Koreans from other studies also showed that the younger age group was associated with fewer subjects meeting the HbA1c target [32], and the present study using KNHANES data revealed that the group under age 40 had significantly more male subjects (S2 Table). Therefore, we expect that once application of the eGFR-based model allows metformin use for more patients, better glycemic control will be attainable in this disease group.

The strength of this study is that we used a comprehensive national data representative of total Korean population. However, it is uncertain whether MDRD and CKD-EPI equations based on a single measurement of serum creatinine accurately represent the actual GFR in Koreans, which may be a limitation of this study. In addition, further studies with other Asian populations are needed to generalize our findings to whole Asian population.

In summary, contrary to our original belief that when measurements of eGFR are used instead of sCr levels in Asians the expansion of the indication for metformin would be minimal or even result in a contraction, the present study confirmed that indications for eGFR-based metformin use will expand in the Korean population. In addition, we could show that currently used sCr-based metformin prescription was indeed a safe measure considering the eGFR category. We believe that the expansion in the indication of metformin prescription will greatly benefit the care and treatment of patients with young-onset diabetes.

## Supporting information

S1 Table MDRD eGFR categories of ineligible and eligible population by the sCr level, NHANES vs KNHANES. (DOCX)

S2 Table Characteristics of young adults with diabetes (20–39 years). (DOCX)
