# Supplementary material for: Effect of prescribing metformin according to eGFR instead of serum creatinine level: A study based on Korean National Health and Nutrition Examination Survey (KNHANES) 2009-2014
Source: PLoS One. 2017 Apr 11;12(4):e0175334. doi: 10.1371/journal.pone.0175334 (PMC5388489; doi:10.1371/journal.pone.0175334)
Supplement: S1 Table — (DOCX) [file pone.0175334.s001.docx]

**S1 Table. MDRD eGFR categories of ineligible and eligible population by the sCr level, NHANES vs KNHANES**

|  | Ineligible by sCr | | | | Eligible by sCr | | | | | |  |
| --- | --- | --- | --- | --- | --- | --- | --- | --- | --- | --- | --- |
|  | sCr ≥1.4 mg/dL for women; ≥1.5 mg/dL for men | | | | sCr <1.4 mg/dL for women; <1.5 mg/dL for men | | | | | |  |
|  | NHANES | | KNHANES | | NHANES | | | KNHANES | | |  |
|  | (7.5% of total adult diabetes)^b^ | | (2.7% of total adult diabetes)^b^ | | (92.5% of total adult diabetes)^b^ | | | (97.3% of total adult diabetes)^b^ | | |  |
| eGFR (mL/min/1.73 m^2^) | Study ^a^ | National estimate ^a^ | Study ^a^ | National estimate ^a^ | | Study ^a^ | National estimate ^a^ | | Study ^a^ | National estimate ^a^ | |
| eGFR<30, n (%) | 122 (35.7) | 444,800 (33.5) | 36 (30.0) | 34,328 (35.6) | | 0 (0.0) | 0 (0.0) | | 0 (0.0) | 0 (0.0) | |
| eGFR≥30, <45, n (%) | 170 (49.7) | 734,900 (55.3) | 68 (56.7) | 46,952 (48.6) | | 53 (1.6) | 271,300 (1.7) | | 27 (0.7) | 16,814 (0.5) | |
| eGFR≥45, n (%) | 50 (14.6) | 148,700 (11.2) | 16 (13.3) | 15,264 (15.8) | | 3,216 (98.4) | 16,037,300 (98.3) | | 3,980 (99.3) | 3,509,468 (99.5) | |
| All, n (%) | 342 (100.0) | 1,328,400 (100.0) | 120 (100.0) | 96,544 (100.0) | | 3,269 (100.0) | 16,308,600 (100.0) | | 4,007 (100.0) | 3,526,282 (100.0) | |
| Expanding population | 50 (14.6) | 148,700 (11.2) | 16 (13.3) | 15,264 (15.8) | | - | - | | - | - | |

Values are presented as n (%).

^a^ “Study” refers to actual study participants’ data. “National estimate” refers to representative population estimates of the total national population.

^b^Percentages presented in this line are used with the national estimates data.
