# Supplementary material for: Effect of prescribing metformin according to eGFR instead of serum creatinine level: A study based on Korean National Health and Nutrition Examination Survey (KNHANES) 2009-2014
Source: PLoS One. 2017 Apr 11;12(4):e0175334. doi: 10.1371/journal.pone.0175334 (PMC5388489; doi:10.1371/journal.pone.0175334)
Supplement: S2 Table — (DOCX) [file pone.0175334.s002.docx]

**S2 Table. Characteristics of young adults with diabetes (20-39 years)**

|  | 20 ≤ age < 40 | 40 ≥ age | *P*^b^ (National estimate) |
| --- | --- | --- | --- |
|  | National estimate^a^ | National estimate^a^ |  |
| N (%) | 278,923 (7.7) | 3,343,903 (92.3) |  |
| Male sex, n (%) | 176,310 (63.2) | 1,846,635 (55.2) | 0.043 |
| Hypertension, n (%) | 63,819 (22.9) | 1,861,533 (55.7) | <0.001 |
| HbA1C < 7% (53 mmol/mol), n (%)^c^ | 129,230 (48.6) | 1,830,212 (55.4) | 0.119 |
| BMI, kg/m^2^ | 27.0 ± 0.41 | 25.2 ± 0.07 | <0.001 |
| MDRD eGFR, mL/min/1.73 m^2^ | 99.5 ± 1.38 | 81.7 ± 0.38 | <0.001 |

Values for categorical variables are presented as n (%); for continuous variables as mean ± standard error (for National estimate data).

^a^“National estimate” refers to representative population estimates of the total Korean population.

^b^Statistical analyses were performed using t-test or χ2 test.

^c^The total population among HbA1C < 7% (53 mmol/mol) is 3,568,887 because of missing HbA1C data.
